# Rate and Extent of Growth of a Model Extremophile, *Archaeoglobus fulgidus*, Under High Hydrostatic Pressures

**DOI:** 10.3389/fmicb.2020.01023

**Published:** 2020-06-12

**Authors:** Gina C. Oliver, Anaïs Cario, Karyn L. Rogers

**Affiliations:** ^1^Department of Earth and Environmental Sciences, Rensselaer Polytechnic Institute, Troy, NY, United States; ^2^Rensselaer Astrobiology Research and Education Center, Rensselaer Polytechnic Institute, Troy, NY, United States

**Keywords:** piezophiles, *Archaeoglobus fulgidus*, high-pressure microbiology, microbial physiology, deep marine biosphere

## Abstract

High hydrostatic pressure (HHP) batch cultivation of a model extremophile, *Archaeoglobus fulgidus* type strain VC-16, was performed to explore how elevated pressures might affect microbial growth and physiology in the deep marine biosphere. Though commonly identified in high-temperature and high-pressure marine environments (up to 2–5 km below sea level, 20–50 MPa pressures), *A. fulgidus* growth at elevated pressure has not been characterized previously. Here, exponential growth of *A. fulgidus* was observed up to 60 MPa when supported by the heterotrophic metabolism of lactate oxidation coupled to sulfate reduction, and up to 40 MPa for autotrophic CO_2_ fixation coupled to thiosulfate reduction via H_2_. Maximum growth rates for this heterotrophic metabolism were observed at 20 MPa, suggesting that *A. fulgidus* is a moderate piezophile under these conditions. However, only piezotolerance was observed for autotrophy, as growth rates remained nearly constant from 0.3 to 40 MPa. Experiments described below show that *A. fulgidus* continues both heterotrophic sulfate reduction and autotrophic thiosulfate reduction nearly unaffected by increasing pressure up to 30 MPa and 40 MPa, respectively. As these pressures encompass a variety of subsurface marine environments, *A. fulgidus* serves as a model extremophile for exploring the effects of elevated pressure on microbial metabolisms in the deep subsurface. Further, these results exemplify the need for high-pressure cultivation of deep-sea and subsurface microorganisms to better reflect *in situ* physiological conditions.

## Introduction

High hydrostatic pressure (HHP) is an inherent characteristic of all deep marine ecosystems (e.g., Jannasch and Taylor, 1984; Oger and Jebbar, 2010; Picard and Daniel, 2013), which are anchored by diverse microbial communities of primary producers and consumers. However, growth physiology under high-pressure conditions for most of the resident species is poorly understood, thus limiting our understanding of how the deep subsurface microbiome cycles mass and energy in its natural habitats. HHP microbial cultivation, though first introduced by Zobell and Oppenheimer (1950), has still not been widely adopted, largely due to costs and complexities of developing specialized high-pressure equipment (e.g., Jebbar et al., 2015; McNichol et al., 2016; Cario et al., 2019; Garel et al., 2019). Therefore, most microorganisms recovered from the deep biosphere are cultivated at ambient or near ambient pressures (i.e., 0.1–0.3 MPa) using traditional batch cultivation techniques, giving little insight into their *in situ* physiologies.

In general, studies of microbial responses to high pressure can be divided into those that focus on the potentially deleterious effects of pressure on surface species, and the adaptation mechanisms of piezotolerant and piezophilic organisms. By definition, those species that are negatively impacted by high-pressure conditions are categorized as piezosensitive, while piezotolerant species are insensitive to high pressure up to a limit; finally piezophiles grow optimally at elevated pressure and some of these are incapable of growth at ambient pressure (obligate piezophiles; e.g., Jannasch and Taylor, 1984; Kato et al., 2008; Fang et al., 2010). While these categories are traditionally applied at the strain level, the pressure response can vary within a strain as other growth conditions vary (e.g., metabolism, temperature, energy supply; Nogi et al., 1998; Zhao et al., 2015).

For organisms inhabiting surface environments, increasing pressures can disrupt cell division, nucleoid structure, rates of DNA replication, RNA synthesis and protein synthesis (Welch et al., 1993; Abe et al., 1999; Abe and Horikoshi, 2001). Also, pressures outside the optimal range induce the synthesis of stress response proteins and chaperone molecules (Welch et al., 1993; Abe et al., 1999; Abe and Horikoshi, 2001). Effects of high-pressure on biochemical pathways and physiology have been reviewed by Jaenicke (1983). The most commonly observed response to pressure in piezotolerant or piezophilic species is a change in the protein profile, often related to regulation of cytochromes, membrane proteins, DNA recombination and repair enzymes, heat shock proteins and chaperone molecules (e.g., Straube et al., 1990; Holden and Baross, 1993, 1995; Hei and Clark, 1994; Michels et al., 1996; Marteinsson et al., 1997; Abe et al., 1999; Sun and Clark, 2001; Boonyaratanakornkit et al., 2006). Changes in both lipid composition and fluidity have also been attributed to high-pressure conditions (Kaneshiro and Clark, 1995; Abe et al., 1999). Genomic adaptations to high-pressure include elongated intergenic spacer regions, increases in RNA copy number, and elongation of the 16S rRNA helices (Vezzi, 2005; Lauro and Bartlett, 2007; Lauro et al., 2007). High-pressure modifications have also been identified in the transcriptome of piezophiles (Vezzi, 2005; Boonyaratanakornkit et al., 2006), and studies of some hyperthermophilic Archaea (e.g., *Thermococcus kodakarensis, Thermococcus barophilus, Pyrococcus yayanosii;* Vannier et al., 2015; Michoud and Jebbar, 2016), indicate that the pressure stress response involves a global scale metabolism modification rather than a classical stress response. More generally, a universal adaptation to HHP or a characteristic suite of “high-pressure” genes or proteins has not been identified so far in piezophiles (Jebbar et al., 2015; Zhang et al., 2015; Charlesworth and Burns, 2016).

In addition to these biochemical and molecular studies, several studies focus on how HHP can impact microbial growth (e.g., Zobell and Oppenheimer, 1950; Yayanos et al., 1982; Jannasch et al., 1992; Bartlett, 2002; Takai et al., 2008; Garel et al., 2019). When considering both isolated species and environmental enrichment experiments, Cario et al. (2019) found that initial habitat depth is correlated with a positive growth response to pressure. Furthermore, responses to elevated pressures are generally species- or strain-specific (e.g., Kato et al., 2008; Fang et al., 2010; Jebbar et al., 2015). Among the thermophiles, various responses have been observed, including (i) increases in temperature optima; (ii) increases in temperature maxima; (iii) increases in growth rate; and (iv) increases in metabolic rate (Abe and Horikoshi, 2001; Scoma et al., 2019), although these responses are not uniform across species. In the cases of *Methanopyrus kandleri*, *Pyrococcus abyssi*, and *P. yayanosii*, optimal growth temperatures, maximum growth temperatures, and maximum growth rates all increased with pressures in excess of 0.1 MPa (Erauso et al., 1993; Marteinsson et al., 1997; Takai et al., 2008; Zeng et al., 2009; Birrien et al., 2011). For some thermophiles maximum temperatures increase with pressure, while optimal temperatures remain unchanged (e.g., Marteinsson et al., 1999), and for others growth rate is the primary parameter impacted by increasing pressure (Bernhardt et al., 1987, 1988; Jannasch et al., 1992; Hei and Clark, 1994; Alain et al., 2002; Takai et al., 2009). Metabolic rates, independent of cell growth rates, have also been enhanced by high pressure (Miller et al., 1988).

These studies highlight the variety of microbial response to high pressure, and specifically the lack of predictability with current data. Therefore, realistic models of deep marine microbial ecosystems require more robust characterizations of microbial physiology at conditions that more accurately reflect *in situ* environments, including elevated pressures. Because microorganisms respond differently to pressure, controlled laboratory growth experiments can be used to isolate specific high-pressure adaptive mechanisms from other environmental parameters. Using well-studied, model organisms to characterize HHP growth can greatly advance our understanding of how microbes grow and transduce energy in subsurface high-pressure conditions.

Here a model extremophile, *Archaeoglobus fulgidus* type strain VC-16, was used to explore the effects of HHP on microbial growth. *A. fulgidus* is a marine hyperthermophilic sulfate-reducing archaeon that has been found in both surface and deep environments (e.g., Stetter et al., 1987; Zellner et al., 1989; Burggraf et al., 1990; Stetter, 1992). *A. fulgidus* is one of the best described species of this genus (Achenbach-Richter et al., 1987; Klenk et al., 1997), as it was the first hyperthermophilic sulfate-reducing archaeon isolated and characterized, and one of the first archaea to have its genome sequenced (Stetter, 1988; Thauer and Kunow, 1995; Klenk et al., 1997; Rabus et al., 2013). *A. fulgidus* has been shown to grow as both a chemoorganoheterotroph and a chemolithoautotroph and can utilize various organic and inorganic carbon compounds (e.g., lactate, formate, fatty acids, *n*-alkenes, and *n*-alkanes, CO_2_, CO) and sulfur compounds (i.e., SO${}_{4}^{2-}$, SO${}_{3}^{2-}$, and S_2_O${}_{3}^{2-}$; Stetter et al., 1987; Stetter, 1988, 1992; Zellner et al., 1989; Burggraf et al., 1990; Hartzell and Reed, 2006; Khelifi et al., 2010, 2014; Hocking et al., 2014). Even though *A. fulgidus* was first isolated from a shallow marine hydrothermal system (Stetter et al., 1987), it is a ubiquitous member of subsurface microbial communities. For example, *A. fulgidus* strains have been isolated from a deep-sea hydrothermal vent (Nakagawa et al., 2005), deep oil reservoirs (Stetter et al., 1993; Beeder et al., 1994; L’Haridon et al., 1995), and deep geothermal wells (Fardeau et al., 2009; Table 1). Also, it has been identified via molecular techniques in various deep-sea vents and shallow terrestrial hot springs (e.g., Takai and Horikoshi, 1999; Nercessian et al., 2003; Schrenk et al., 2003; Table 1). Because sulfate reducing archaea play an important role in the biogeochemical cycling of sulfur and carbon (Hartzell and Reed, 2006; Rabus et al., 2013), it is critical to cultivate organisms such as *A. fulgidus* at elevated pressures to assess their ability to cycle carbon and sulfur compounds in deep environments as efficiently as they do at surface pressure conditions.

**TABLE 1:** *Archaeoglobus* Isolates.

Species, Strain	Environment	Depth	Location	Reference
*A. fulgidus*				
VC-16†	Shallow vent	1–10 m	Vulcano, Italy	Stetter et al., 1987
Z	Shallow vent	1–10 m	Vulcano, Italy	Zellner et al., 1989
NS70-A	Deep-sea vent	∼968 m	Iheya North Fields	Nakagawa et al., 2005
TF2	Oil reservoir	2–4 km	North Sea	Stetter et al., 1993
7324	Oil reservoir	2–4 km	North Sea	Beeder et al., 1994
SL5	Oil reservoir	1.6 km	Paris Basin, France	L’Haridon et al., 1995
L3 and L4	Geothermal system	1.9 km	Paris Basin, France	Fardeau et al., 2009
*A. profundus*				
AV18	Deep-sea vent	2 km	Guaymas Basin, Mexico	Burggraf et al., 1990
NI85-A	Deep-sea vent	∼968 m	Iheya North Fields	Nakagawa et al., 2005
*A. veneficus*	Deep-sea vent	3.5 km	Mid-Atlantic Ridge	Huber et al., 1997
*A. infectus*	Deep-sea vent	1.4 km	Izu-Bonin Arc	Mori et al., 2008
*A. sulfaticallidus* PM70-1	Black rust (borehole)	2.65 km	Juan de Fuca Ridge	Steinsbu et al., 2010
*‘A. lithotrophicus’*	Oil reservoir	2–4 km	North Sea	Stetter et al., 1993

*†Type Stain (DSM 4304).*

The common occurrence of *A. fulgidus* in high-pressure subsurface environments suggests that *A. fulgidus* is at least piezotolerant. Growth up to 35 MPa has been reported for a related strain, *A. fulgidus* TF2 (Stetter et al., 1993). Furthermore, in Guaymas Basin hydrothermal sediments, where members of the *Archaeoglobales* have been identified with 16S amplicon sequencing (Teske et al., 2002), radiotracer incubation experiments suggested that microbial sulfate reduction is enhanced by HHP (Kallmeyer et al., 2003). Nonetheless, the pressure-dependent growth of *A. fulgidus* type strain VC-16 remains uncharacterized and the impact that this ubiquitous model hyperthermophile might have on metabolic and biogeochemical networks in high-pressure subsurface environments is unknown. Using a model strain that is metabolically versatile further opens the opportunity to compare heterotrophy to autotrophy within a single species at high pressure, which has yet to be demonstrated. Here the potential for *A. fulgidus* type strain VC-16 to grow at elevated pressures was investigated at pressures up to 70 MPa. Growth rates and cell densities for batch culture growth experiments are reported for both a chemoorganoheterotrophic metabolism coupling lactate oxidation with sulfate reduction, and a chemolithoautotrophic metabolism in which thiosulfate is reduced to H_2_S with H_2_.

## Materials and Methods

### Growth Media Preparation

*Archaeoglobus fulgidus* type strain VC-16 (DSM 4304) was obtained from the Deutsche Sammlung von Mikroorganismen und Zellkulturen GmbH (DSMZ, Braunschweig, Germany). Here, chemoorganoheterotrophic growth experiments contained lactate as the electron donor and carbon source coupled to sulfate reduction. The heterotrophic growth medium contained a sea salt base with a composition as follows (per liter): 0.34g KCl, 15.142g MgSO_4_⋅7H_2_O, 2.75g MgCl⋅6H_2_O, 0.25g NH_2_Cl, 0.056g CaCl_2_⋅2H_2_O, 0.0137g K_2_HPO_4_⋅3H_2_O, 17.8g NaCl, 0.0039g Fe(NH_4_)_2_(SO_4_)⋅6H_2_O, and 1 mL of Wolfe’s trace element solution (Hartzell and Reed, 2006). This sea salt base along with 0.1 mL Resazurin (0.1% solution) was autoclaved at 121°C for 20 min and cooled to room temperature. Subsequently, stock solutions of sodium L-lactate (NaC_3_H_5_O_3_; 2.1 g/L final concentration), yeast extract (1 g/L final concentration), and PIPES (piperazine-*N*,*N*’-bis[2-ethanesulfonic acid]; 3.36 g/L final concentration) was added by filter sterilization in a biosafety cabinet and the pH was adjusted to 6.7. The sterile medium was aseptically distributed into sterile Balch tubes (10 mL of medium), capped with sterile butyl stoppers and crimped. Prior to inoculation, the headspace above the medium was replaced with N_2_ by flushing Balch tubes for 15–20 min and a 2.5% (w/v) Na_2_S⋅9H_2_O stock solution was added to a final concentration of 1 mM to obtain anoxic conditions (Cario et al., 2016).

Here, chemolithoautotrophic growth was measured for thiosulfate reduction with H_2_ supporting CO_2_ fixation (Stetter, 1988; Hartzell and Reed, 2006; Hocking et al., 2014) after *A. fulgidus* VC-16 was adapted from a heterotrophic metabolism to an autotrophic metabolism (see Supplementary Material for details). The autotrophic growth medium was similar to the heterotrophic medium with the following modifications: MgSO_4_⋅7H_2_O, sodium L-lactate, and yeast extract were omitted; MgCl⋅6H_2_O was increased to 6.38 g/L to maintain salinity; CaCl_2_⋅2H_2_O was increased to 0.14 g/L; PIPES was replaced with 2 g/L NaHCO_3_. To obtain anoxia, the medium was boiled under an N_2_ atmosphere and the pH was adjusted to 7.3 to compensate for the decrease in pH induced by CO_2_ addition. The anoxic, pH-adjusted medium was transferred to N_2_-flushed serum bottles, sealed with butyl rubber stoppers and crimp-sealed prior to autoclaving. Following sterilization, the headspace was vacuumed and flushed with a 80% H_2_: 20% CO_2_ gas mixture. Prior to inoculation, 2.5% (w/v) Na_2_S⋅9H_2_O was added to a final concentration of 1 mM to obtain anoxic conditions (Cario et al., 2016), and subsequently each serum bottle was amended with a sterile, anoxic 2.5% (w/w) Na_2_S_2_O_3_ stock solution to a final concentration of 47 mM (Hocking et al., 2014).

### Heterotrophic Ambient Pressure and HHP Batch Cultivation Experiments

**FIGURE 1:**
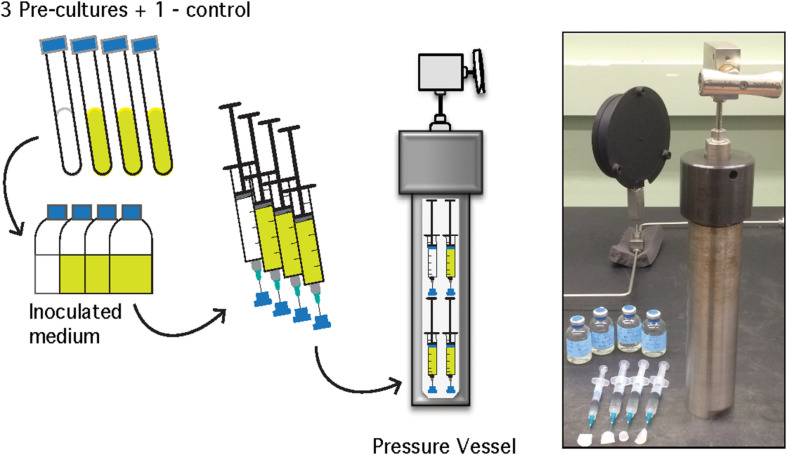
Schematic of *A. fulgidus* HHP heterotrophic batch culture growth experiments (performed in triplicate with a negative control). See text for details.

For HHP batch cultivation, triplicate batch culture experiments and a single uninoculated control were conducted at 0.1–70 MPa at 10 MPa increments. For each triplicate, 30 mL of sterile, anaerobic medium was inoculated with 3% (v/v) logarithmic phase *A. fulgidus* cells from three separate precultures to a final cell concentration of ∼1.25 × 10^7^ cells/mL. Inoculated growth medium for each triplicate was transferred aseptically into 5 mL, N_2_-flushed plastic syringes (BD medical). After transfer, excess N_2_ gas was expelled from each syringe before the needle was embedded into a silicone stopper to maintain a closed system. The syringes were placed inside pre-heated stainless steel pressure vessels, filled with deionized water, and pressurized using a hydraulic screw pump (High Pressure Equipment Company^©^) to the desired pressure (Figure 1; Zobell and Oppenheimer, 1950; Yayanos, 2001). Parallel, ambient pressure experiments were carried out in Balch tubes (Balch et al., 1979), or in headspace-free syringes similar to HHP experiments without pressurization (0.1 MPa). Experiments conducted in Balch tubes were amended with a 0.3 MPa N_2_ headspace following inoculation. All experiments were incubated at 83°C for the duration of each experiment. Each HHP batch culture experiment, along with the ambient-pressure controls were subsampled ∼11 times to obtain complete growth curves. Subsamples for cellular enumeration were taken every 2–4 h after inoculation for 36 h for 0.1–40 MPa experiments and up to 52 h for 50–70 MPa experiments. Pressure vessels were decompressed at an average rate of 19 MPa/min and 0.5 mL subsamples were fixed with 2.5% (v/v) gludaraldehyde for subsequent enumeration.

Because the static pressure vessels require decompression for subsampling and such decompression can negatively impact microbial growth during high-pressure cultivation (Park and Clark, 2002), a suite of high-pressure experiments tested the effects of multiple decompression-repressurization cycles on growth (Supplementary Figure 1). In order to minimize the effects of decompression cycles four replicates of each experiment (triplicate growth experiments and one uninoculated control) were distributed across four static pressure vessels and subsampling alternated among these. Therefore, a total of twelve 5 mL syringe cultures, four syringes from each triplicate for each vessel, and four 3 mL syringes filled with uninoculated medium for negative controls, were prepared for each HHP experiment. To obtain complete growth curves (36–52 h), each vessel was subjected to a maximum of four decompression-repressurization cycles, with at least 6 h between cycles.

Lastly, *A. fulgidus* cell recovery after a 52-h exposure at 70 MPa for the heterotrophic metabolism was tested. Logarithmic-phase *A. fulgidus* cells were incubated in triplicate at 70 MPa and 83°C for 52 h in a single, heated pressure vessel. Because the effects of decompression on *A. fulgidus* cells at 70 MPa were unknown, these incubations were also decompressed and repressurized four times at 18, 24, 36, and 52 h after inoculation. Following 52 h of incubation, a final concentration of ∼1.1 × 10^7^ cells/mL was transferred into sterile anaerobic medium in Balch tubes at 0.3 MPa and growth was monitored visually until the culture became turbid after 64 h and direct counts of triplicate experiments were taken.

### Autotrophic Ambient Pressure and HHP Batch Cultivation Experiments

**FIGURE 2:**
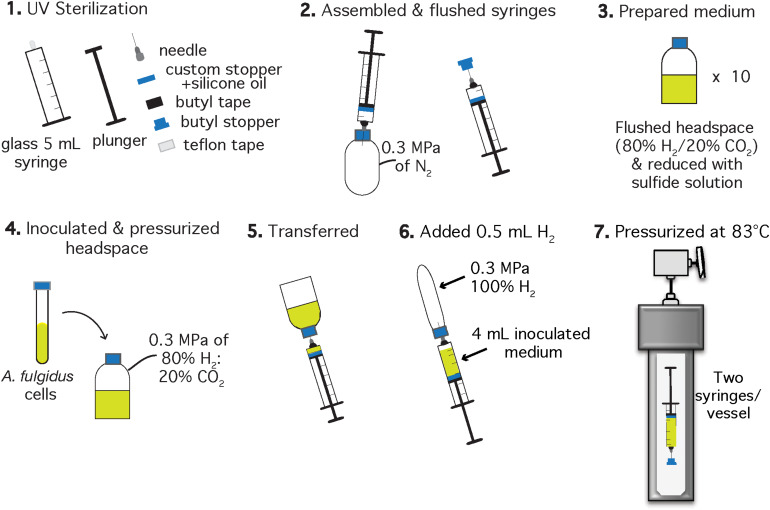
Schematic of the experimental setup for *A. fulgidus* HHP autotrophic growth experiments after Takai et al. (2008). Each experiment was performed in triplicate with a negative control. Two syringes with inoculated growth medium were incubated in one high pressure vessel and one vessel contained one growth syringe and one negative control.

Similar to HHP heterotrophic growth experiments, HHP experiments exploring autotrophy were also conducted in syringes contained in static pressure vessels, with replicates distributed to minimize decompression-repressurization cycles. However, because the autotrophic metabolism depends on significant concentrations of dissolved volatiles (H_2_, CO_2_), modifications to the standard protocol were necessary. Specifically, all HHP autotrophic experiments were carried out in glass, gas-tight syringes and additional H_2_ was added prior to pressurization to mitigate gas loss. The detailed experimental protocol is outlined in Figure 2 and described below.

Autotrophic HHP batch cultivation of *A. fulgidus* was carried out in glass gas-tight syringes (Hamilton, 5 mL luer slip tip syringes) following methods of Takai et al. (2008) and Tasumi et al. (2015). Clean and dried 5 mL glass syringes (Hamilton, Reno, NV, United States), chromatography needles, butyl stoppers, Teflon tape, and butyl rubber tape for sealing the needle-syringe connection, were sterilized under UV light for 1 h in a biosafety cabinet (Figure 2, Step 1). Syringes were assembled to maintain gas-tight conditions during incubation by wrapping the syringe luer slip tip with Teflon, then attaching the needle to the syringe and finally sealing the needle-syringe connection with butyl rubber tape. Syringes and attached needles were then flushed with N_2_ using an over-pressurized (0.3 MPa) bottle (Figure 2, Step 2). The autotrophic, anaerobic growth medium was prepared in 30 mL serum bottles (Figure 2, Step 3). Each serum bottle was inoculated with 1.5% (v/v) logarithmic phase *A. fulgidus* cells from three separate precultures to a final cell concentration of ∼3 × 10^6^ cells/mL. The headspace was replaced with a 80% H_2_: 20% CO_2_ gas mixture to 0.3 MPa and left to equilibrate for ∼5 min (Figure 2, Step 4). Next, 4 mL of inoculated and gas-equilibrated medium was transferred into each syringe from a serum bottle (Figure 2, Step 5). Previous experiments containing 3 mL ultrapure water (18.2 MΩ) and 1 mL H_2_ in gas-tight glass syringes showed at most a 0.2 mL H_2_ loss (by volume) after incubation at 60 MPa and 83°C for 150 h. Therefore, ∼0.5 mL of 100% H_2_ was added to each syringe to mitigate H_2_ loss on pressurization (Figure 2, Step 6), thus making these experimental conditions more comparable to experiments carried out in Balch tubes with a 0.3 MPa 80% H_2_/20% CO_2_ headspace. Finally, the needles were embedded into butyl rubber stoppers to maintain a closed system. All of the syringes were then placed into pre-heated 83°C vessels and pressurized as described in section “Heterotrophic Ambient Pressure and HHP Batch Cultivation Experiments” (Figure 2, Step 7).

Two near-ambient pressure control experiments were conducted in serum bottles. First, the inoculated medium remaining in the serum bottles used to fill the glass syringes was incubated at 83°C to monitor growth under optimum conditions. Second, to observe if there was a significant difference in growth using traditional batch culture techniques versus HHP techniques at 0.3 MPa, *A. fulgidus* growth in serum bottles with a ∼15 mL headspace was compared to *A. fulgidus* growth in 5 mL glass syringes with ∼0.5 mL gas phase incubated in the static pressure vessels. At these pressures a two-phase system is maintained in each experiment (serum bottle or glass syringe), and both phases achieve equivalent pressures. To achieve such low pressures in these vessels, an additional pressure gauge was placed onto the pressure line. This gauge was rated up to 0.7 MPa so that 0.3 MPa could be reached more precisely. Each serum bottle was subsampled six times during the course of each experiment.

*Archaeoglobus fulgidus* autotrophic HHP batch culture experiments were performed from 0.3 to 60 MPa in ∼20 MPa increments. Each HHP batch culture experiment was performed in triplicate with a negative control, along with the ambient-pressure controls described above. Five static high-pressure vessels, containing two 5 mL glass syringes (one experiment and a negative control), were pressurized and decompressed only once for sampling to minimize decompression-repressurization cycles. Vessels were decompressed at an average rate of 19 MPa/min. To obtain growth curves through the stationary phase, five 0.5 mL subsamples were taken over ∼145–215 h, fixed with 2.5% (v/v) gludaraldehyde, and later enumerated via light microscopy. Six subsamples were taken from experiments conducted in serum bottles.

### Direct Cell Counts

For heterotrophic growth, cell densities were estimated by direct counting of cells fixed in 2.5% (v/v) gludaraldehyde filtered onto 0.2 μm black polycarbonate membranes (EMD Millipore) and stained with DAPI (4′,6-diamidino-2-phenylindole dihydrochloride, Sigma Aldrich; Kepner and Pratt, 1994), under 200x magnification on an Olympus XM10 fluorescence microscope. For autotrophic growth, cell density was estimated by direct counts from cells fixed in 2.5% (v/v) gludaraldehyde on a Thoma-chamber (depth: 0.02 mm; Brand, Wertheim, Germany) using a light microscope (model XM: Olympus) under 80x magnification (e.g., Huber et al., 1989; Hei and Clark, 1994; Blöchl et al., 1997; Cario et al., 2016). To ensure consistency between the two counting methods, cells were enumerated in triplicate experiments by both DAPI and using a Thoma-chamber (depth: 0.02 mm; Brand, Wertheim, Germany). Cell counts for each method were comparable (Supplementary Figure 2). Maximum cell densities were observed from stationary phase for all pressure conditions and error bars indicate standard deviation from at least triplicate experiments. Growth rates were calculated from the logarithmic growth phase slopes from triplicate batch culture experiments using the LINEST function in Excel. Error bars indicate the standard error from linear regressions of triplicate experiments.

## Results

**FIGURE 3:**
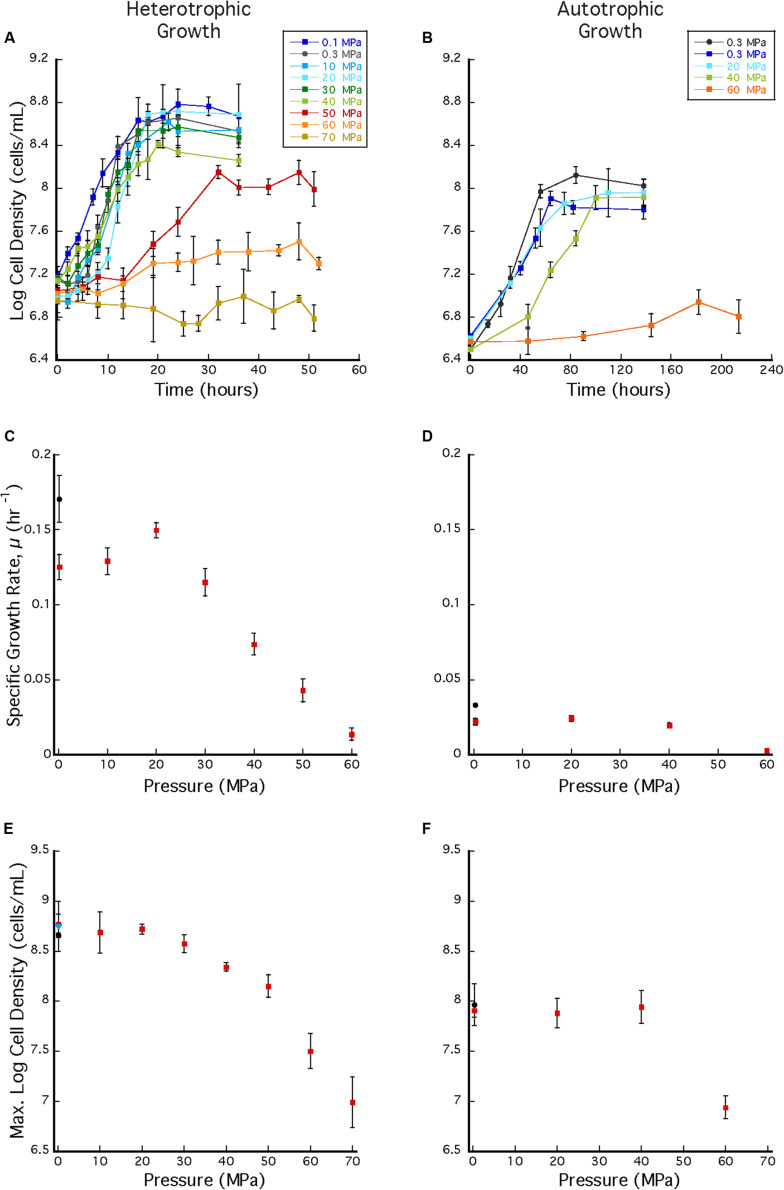
**(A)** *A. fulgidus* heterotrophic HHP growth curves from 0.1 to 70 MPa in syringes (colored squares) and Balch tubes at 0.3 MPa (black circles). **(B)** *A. fulgidus* autotrophic HHP growth curves from 0.3 to 60 MPa in glass syringes (colored squares) and in serum bottles (black circles). **(C)** *A. fulgidus* HHP heterotrophic growth rates from 0.1 to 70 MPa in syringes (red squares) and at 0.3 MPa in Balch tubes (black circle) calculated from observed growth curves **(A)**. **(D)** *A. fulgidus* HHP autotrophic growth rates from 0.3 to 60 MPa in syringes (red squares) and at 0.3 MPa in serum bottles (black circle) calculated from growth curves **(B)**. Growth rates were calculated from 3 to 5 data points measured from exponential growth from at least triplicate experiments. *A. fulgidus* HHP heterotrophic **(E)** and autotrophic **(F)** maximum cell densities measured when grown in syringes (red squares), Balch tubes or serum bottles (black circles), and at 0.3 MPa after exposure to 70 MPa [blue triangle in **(A)**]. No growth was observed at 70 MPa. Error bars represent the standard deviation from at least triplicate experiments.

**FIGURE 4:**
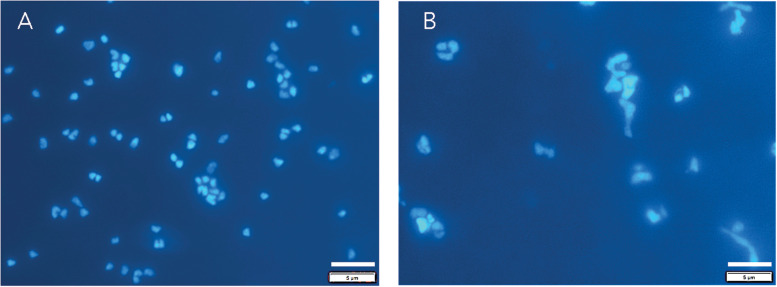
DAPI stained *A. fulgidus* cells grown in a heterotrophic medium after 24 h of growth at 0.1 MPa **(A)** in Balch tubes and at 50 MPa **(B)** in syringes. Dilution factors for were 20x **(A)** and 4x **(B)**. Bar is 5 μm.

*Archaeoglobus fulgidus* grew in a heterotrophic growth medium from 0.1 to 60 MPa under HHP batch cultivation at 83°C (Figure 3A). Standard growth curves show similar growth patterns and cell densities up to 30 MPa, while slower growth was observed from 40 to 60 MPa; no notable increases in cell densities were measured at 70 MPa (Figure 3A). Growth rates increased from 0.1 to 20 MPa and decreased from 20 to 60 MPa (Figure 3C). Maximum cell densities were similar from 0.1 to 30 MPa and decreased with pressures above 30 MPa (Figure 3E). The maximum growth rate for experiments that used the syringe-based HHP techniques (e.g., syringes without headspace) was observed at 20 MPa (0.15 ± 0.005 hr^–1^, Figure 3C), while the growth rate at 0.1 MPa was 0.13 ± 0.008 hr^–1^ (Figure 3C), suggesting that *A. fulgidus* is a moderate piezophile under these conditions. Growth rates and maximum cell densities of *A. fulgidus* began to decline at 30 MPa and 40 MPa, respectively (Figures 3C,E), and growth was barely detectable at 60 MPa (0.014 ± 0.004 hr^–1^; Figures 3C,E). No growth was measured at 70 MPa and the maximum cell density (9.82 ± 1.78 × 10^6^ cells/mL, Figures 3A,E) was only slightly higher than initial values (9.04 ± 1.3 × 10^6^ cells/mL, Figure 3E). Even though growth did not occur at 70 MPa, cells exposed to 70 MPa for 52 h that were subsequently transferred to 0.3 MPa recovered to high cells densities (5.6 ± 0.25 × 10^8^ cells/mL) after 64 h of growth at 0.3 MPa (Figure 3E). *A. fulgidus* cells are normally coccoid but at growth pressures above 40 MPa, cell morphology was noticeably different (Figure 4). Cells that are normally coccoidal (Figure 4A) became irregular and elongated at 50 MPa and 60 MPa (Figure 4B). Similar morphological changes have been observed in other strains under HHP stress (e.g., Zobell and Oppenheimer, 1950; Donaldson et al., 1989).

In addition to the HHP batch cultivation techniques that employed headspace-free, stoppered syringes, growth experiments were also conducted in Balch tubes with a ∼17 mL gas headspace. These experiments were conducted at 0.3 MPa and compared to experiments incubated at 0.3 MPa in headspace-free syringes. In the latter experiments cell clustering was observed over a wide pressure range (0.1–50 MPa), while aggregation was absent in Balch tubes (Supplementary Figure 3). Growth rates at 0.3 MPa were also significantly different between the two techniques, and the overall highest growth rate (0.17 ± 0.015 hr^–1^; Figure 3C black circle) was observed in Balch tubes at near-ambient pressure conditions. Nonetheless, these experiments have no HHP counterpart and cannot be directly compared to growth in syringes.

*Archaeoglobus fulgidus* grew autotrophically from 0.3 to 40 MPa at 83°C under HHP batch cultivation (Figure 3B). Similar growth patterns were observed from 0.3 to 40 MPa, though growth at 40 MPa had a longer lag phase. Only slight growth was observed at 60 MPa (Figure 3B). *A. fulgidus* was grown autotrophically at 0.3 MPa in serum bottles with a headspace ∼38% by volume, and also grown using HHP methods in syringes with minimal headspace of ∼0.5 mL of H_2_ at 0.3 MPa and no headspace from 10 to 60 MPa. For autotrophic growth experiments, similar growth rates were observed from 0.3 to 40 MPa (0.022 ± 0.002 hr^–1^, 0.0241 ± 0.002 hr^–1^, and 0.0195 ± 0.002 hr^–1^, respectively), suggesting that *A. fulgidus* is a piezotolerant autotroph (Figure 3D). Slightly higher growth rates were observed in experiments conducted in serum bottles at 0.3 MPa (0.0329 ± 0.001 hr^–1^; Figure 3D black circle), likely due to the presence of an H_2_/CO_2_ gas phase. Again, cultivation in serum bottles at 0.3 MPa is not directly comparable to growth in syringes under HHP culture conditions. For HHP batch cultivation, similar maximum cell densities were measured at 0.3–40 MPa (9.22 ± 1.33 × 10^7^ cells/mL – 1.38 ± 0.215 × 10^8^ cells/mL; Figure 3F). Minor growth was measured at 60 MPa with an average maximum cell density that reached 8.67 ± 1.29 × 10^6^ cells/mL from an average inoculum cell density of 3.16 ± 1.65 × 10^6^ cells/mL (Figure 3F). As expected, autotrophic growth was slower and had lower cell densities compared to heterotrophic growth (Prieur and Marteinsson, 1998). Nevertheless, these results indicate that *A. fulgidus* is a piezotolerant autotroph up to 40 MPa.

**FIGURE 5:**
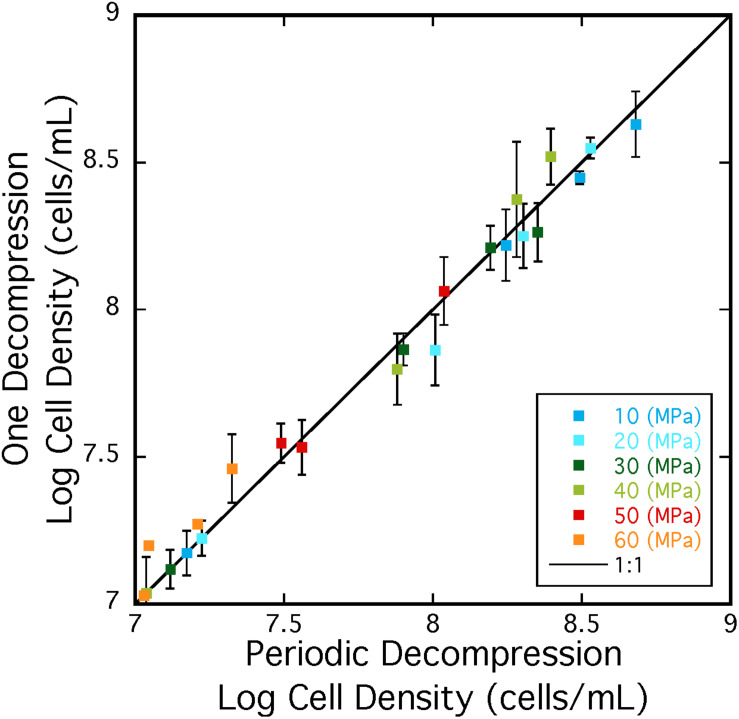
Cell densities *A. fulgidus* grown in a heterotrophic medium, and exposed to one cycle (*y*-axis) or multiple cycles of decompression/repressurization (*x*-axis) from 10 to 60 MPa. The 1:1 correspondence is shown as a black line. Best-fit lines and respective *R*-values are; *y* = –0.23784 + 0.96712x *R* = 0.999 (10 MPa), *y* = –0.011249 + 0.99586x *R* = 0.991 (20 MPa), *y* = –0.29188 + 0.95957x *R* = 0.996 (30 MPa), *y* = –0.65328 + 1.087x *R* = 0.993 (40 MPa), *y* = –0.014095 + 1.0042x *R* = 0.990 (50 MPa), *y* = –1.1686 + 31.1757x *R* = 0.928 (60 MPa). Error bars are deviations from the average of triplicate experiments.

Repeated cycles of decompression and repressurization did not significantly impact heterotrophic growth of *A. fulgidus* from 10 to 50 MPa. Figure 5 shows that cell density measurements in experiments with only one decompression/repressurization cycle were similar to those with multiple cycles. Slightly higher growth densities at 10–30 MPa for cells decompressed multiple times versus once might suggest that repressurization may have a positive impact on growth (Yayanos, 1995). Decompression may have had a negative impact on cells decompressed multiple times at 60 MPa, as maximum cell densities at 60 MPa for cells decompressed once were 3.02 ± 0.10 × 10^7^ cells/mL and those decompressed multiple times were 2.15 ± 0.59 × 10^7^ cells/mL. However, low growth yields at 60 MPa contribute to a low *R*-value, making it difficult to discern any affects from subsampling decompression. Nevertheless, these results confirm high-pressure growth of *A. fulgidus* up to 50 MPa with cellular viability unaffected by periodic decompressions.

*Archaeoglobus fulgidus* (type strain VC16) is capable of growing at HHP conditions both heterotrophically and autotrophically and displays both piezophilic and piezotolerant behavior depending on metabolic strategy. Growing heterotrophically, *A. fulgidus* is a moderate piezophile, with maximum specific growth rates measured at 20 MPa for the cultivation conditions described here. On the other hand, *A. fulgidus* growth rates and stationary-phase cell densities were similar for autotrophy from 0.3 to 40 MPa, indicating only piezotolerance for these autotrophic conditions. Interestingly, growth rates under traditional batch culture generated different results from the HHP syringe-based techniques at 0.1 MPa or 0.3 MPa for both metabolisms, highlighting that different cultivation methods can impact microbial growth patterns. Additionally, cell clustering in experiments using the HHP batch cultivation technique was observed over most of the pressure range (0.1–50 MPa). The cause for cell clustering was not resolved during this study, however, additional analyses are underway to investigate this physiological response. Taken together, these results suggest that *A. fulgidus* is capable of both heterotrophic and autotrophic growth in high-pressure, subsurface, marine environments.

## Discussion

*Archaeoglobus fulgidus* type strain VC-16 was originally isolated from a shallow marine hydrothermal system, and its discovery was notable as this species was the only archaeal sulfate reducer known at that time (Stetter et al., 1987, 1990). Initially this species was thought to be the “shallow”-dwelling *Archaeoglobus* analog to *Archaeoglobus profundus*, isolated from the hot sediments of Guaymas Basin at ∼2 km depth (∼20 MPa; Burggraf et al., 1990). Yet since its discovery, several other *A. fulgidus* strains have been isolated from various deep environments (Table 1), expanding the habitat range for this species. These include three strains isolated from multiple oil reservoirs at depths of up to 4 km (corresponding to ∼40 MPa hydrostatic pressure and even higher lithostatic pressures; Stetter et al., 1993; Beeder et al., 1994; L’Haridon et al., 1995), and two additional *A. fulgidus* strains isolated from the Paris Basin at 1.9 km (Fardeau et al., 2009). Furthermore, this species is commonly identified in various mid-ocean ridge vent systems including the Mid-Atlantic Ridge, the Juan de Fuca Ridge, and the East Pacific Rise (e.g., Reysenbach et al., 1998, 2000; Schrenk et al., 2003), with depths between ∼2 and 3.5 km (∼20–35 MPa). Several other *Archaeoglobus* species have been isolated from various subsurface environments (Table 1), confirming the ubiquity of this genus in high-pressure, subsurface ecosystems. As most of the sampling, analysis and enrichment techniques applied to samples from these high-pressure environments usually involves sample decompression (e.g., Jannasch and Wirsen, 1973; Bianchi et al., 1999; Peoples and Bartlett, 2017; Cario et al., 2019), recovery of this and related species is consistent with the results reported here showing that *A. fulgidus* is largely unaffected by decompression and repressurization (Figure 5). Other strains that are sensitive to decompression are likely excluded from similar analyses via decompression sample bias.

This widespread distribution of *Archaeoglobus* species in subsurface high-pressure environments stands in contrast to the paucity of evidence confirming their growth at elevated pressures. The only other reported HHP growth experiments performed on *A. fulgidus* was strain TF2 (Stetter et al., 1993), which was isolated from a ∼3 km deep oil reservoir and reported to grow at 42 MPa on crude oil as a sole growth substrate (Stetter et al., 1993), reflecting its adaptation to its immediate habitat. Also, an archaeal strain (L3), most closely related to *A. fulgidus*, was isolated from a 2 km geothermal well and was successfully cultivated at ∼0.2 MPa under autotrophic conditions (H_2_ and CO_2_ as a sole carbon source in the presence of thiosulfate; Fardeau et al., 2009). Strain L3 exhibited several phenotypic differences from the *A. fulgidus* type strain VC-16 and required yeast extract for growth (Fardeau et al., 2009). In contrast, the autotrophic HHP experiments reported here for type strain VC-16 extend the autotrophic pressure range for growth to 40 MPa, without supplemental vitamins or yeast extract. These differences once again highlight the significant differences in physiological response to elevated pressure, even within a species.

Consistent with the widespread distribution of *Archaeoglobus* species in high-pressure environments, the experiments reported here show that the *A. fulgidus* type strain VC-16 is moderately piezophilic for chemoorganoheterotrophy, and piezotolerant for chemolithoautotrophy. This adds to the currently identified 58 characterized piezotolerant and piezophilic microorganisms (Jebbar et al., 2015; reviewed in Fichtel et al., 2015; Dalmasso et al., 2016; Kusube et al., 2017; Liu et al., 2018; Cario et al., 2019; Yu et al., 2019), a majority of which (52) are chemoorganoheterotrophs. In the HHP experiments reported here, the physiologic responses of *A. fulgidus* depended primarily on metabolic strategy. Under autotrophic conditions, the type strain was piezotolerant with similar growth rates at 0.3 and 20 MPa. In contrast, *A. fulgidus* VC-16 growing heterotrophically was a moderate piezophile with a maximum growth rate at 20 MPa. Other studies have shown both piezotolerant and piezophilic behaviors within a single species, but these variations have more commonly corresponded to temperature differences. For example, Nogi et al. (1998) found that *Moritella japonica* grew optimally as a piezophile at 15°C and 50 MPa but was piezotolerant at 10°C, with an optimum growth pressure at 0.1 MPa. Similarly, the hyperthermophilic archaeon, *Thermococcus eurythermalis*, exhibited piezophily at 95°C, but was more piezotolerant at 85°C (Zhao et al., 2015). All of the HHP experiments described here were carried out at 83°C, this strain’s optimum temperature at 0.1 MPa (Stetter, 1988), thus it remains unknown if *A. fulgidus’* piezophily is temperature dependent. To our knowledge, HHP growth for both heterotrophy and autotrophy within a single species has not been characterized previously (see Jebbar et al., 2015 for a review). The thermophilic piezophile, *Piezobacter thermophilus*, was shown to be a strict piezophile under autotrophic conditions, and while this species did utilize organic compounds at ambient pressures, HHP organotrophy was not confirmed (Takai et al., 2009). Furthermore, several mesophilic sulfate reducers are known to grow both autotrophically and heterotrophically (e.g., *Desulfobacterium autotrophicum*, *Desulfobacterium vacuolatum*, *Desulfobacter hydrogenophilus*; Brysch et al., 1987), but their ability to grow autotrophically and heterotrophically at high pressure is unknown since only a handful of mesophilic sulfate reducers have been grown at pressure (e.g., Bale et al., 1997; Alazard et al., 2003; Khelaifia et al., 2011; Williamson et al., 2018). As *A. fulgidus* employs both metabolic strategies at elevated pressure, this strain could serve as a model to further explore the enzymatic and bioenergetic distinctions between heterotrophy and autotrophy at elevated pressures.

The increased growth rates of *A. fulgidus* carrying out heterotrophic sulfate reduction at elevated pressures suggest an important role for this hyperthermophilic sulfate reducer in high-temperature, subsurface environments. In general, heterotrophic sulfate reduction is a key metabolism in a variety of anoxic, organic-rich environments. In marine sediments, sulfate reduction can significantly impact sedimentary geochemical profiles (Goldhaber, 2003), and sulfate reduction and anaerobic oxidation of methane (AOM) dominate throughout methane rich marine sediments and play an important role in global carbon cycling (Hinrichs and Boetius, 2002; Joye et al., 2004; Meulepas et al., 2010). In subsurface oil reservoirs, sulfate reduction is the main contributor to crude oil degradation, negatively impacting both economic and safety factors (e.g., Gittel et al., 2009; Gieg et al., 2010, 2011). The importance of *Archaeoglobus* species in these subsurface hydrothermal environments has always been inferred from its ubiquity in phylogenetic diversity studies of these ecosystems, but the specifics of its high-pressure metabolism have been unknown. As the first isolated sulfate-reducing archaeon (Stetter, 1988; Thauer and Kunow, 1995; Rabus et al., 2013), *A. fulgidus* and other members of this genus were recognized as filling a specific ecological niche in hot, sulfate-rich, anoxic surface marine environments. With optimum growth temperatures between 75 and 90°C, these hyperthermophilic sulfate reducing archaea thrive between the thermophilic sulfate reducing bacteria (optimum growth temperatures typically between 40 and 70°C; Rabus et al., 2013) and the hyperthermophilic sulfur metabolizing archaea (optimum growth temperatures typically between ∼70 and 100°C; Reysenbach et al., 2000; Huber et al., 2006; Hartzell and Reed, 2006). The ubiquity of *Archaeoglobus* species in the subsurface suggests that they play a similar role in subsurface environments.

Furthermore, recent reports suggest that both elevated temperature and elevated pressure are critical parameters constraining the ecological niche that sulfate reducers occupy in hydrothermal sediments. For example, in Guaymas Basin, Kallmeyer and Boetius (2004) found that the highest sulfate reduction rates occurred at elevated pressures (22–45 MPa) and at higher temperatures than AOM, suggesting that sulfate reduction is mainly responsible for carbon cycling in higher temperature sediments, and that sulfate reduction rates are pressure-dependent. As a hyperthermophile that can grow up to 95°C (Hartzell et al., 1999), *A. fulgidus* VC-16 could cycle carbon in this high-temperature niche, and the results described here expand the habitat of *A. fulgidus* to include high-pressure environments. Our results expand the pressure range of *A. fulgidus* VC-16 to 0.1–60 MPa for heterotrophic metabolisms and 0.1–40 MPa for autotrophy. These pressures encompass the average ocean floor depth, 3.688 km (i.e., ∼37 MPa) and associated hydrothermal marine sediments, depths of many oil field reservoirs ranging from ∼1-3 km depth (Pinder, 2001), and the average depth of black smoker hydrothermal vents at ∼2.1 km (i.e., ∼21 MPa), and even the deepest known hydrothermal system, the Mid Cayman Rise, where members of the *Archaeoglobales* have been identified with 16S amplicon sequencing (Reveillaud et al., 2016). With the ability to carry out heterotrophic sulfate reduction at both high-temperature and high-pressure conditions, *A. fulgidus* likely plays a significant role in cycling carbon and sulfur in these environments.

## Conclusion

Overall, the high-pressure growth of *A. fulgidus* is unsurprising for its consistency with observations of this species throughout both shallow- and deep-marine hydrothermal systems. However, the expansive pressure range and metabolic variability across that pressure range once again underscores that this species and others within this genus require further investigation, and can serve as model organisms to better understand microbial physiology in hydrothermal environments. *A. fulgidus* would be an ideal subject for a vast array of analyses (molecular and biogeochemical) over a range of pressure conditions. For instance, experiments to explore membrane structure and functionality under different pressure, decompression, and metabolic conditions may lend some insight into this species’ ability to adapt to both surface and subsurface environments. Additionally, other *Archaeoglobus* species that have been isolated from high-pressure environments (Table 1) are likely also piezotolerant and piezophilic. Comparison across these species at both ambient and HHP conditions, especially focusing on comparative transcriptomics and proteomics, could reveal mechanisms of pressure adaptation. Finally, as a majority of bacterial and archaeal cells inhabit high-pressure environments (e.g., Whitman et al., 1998; Jebbar et al., 2015; Bar-On et al., 2018), further HHP batch cultivation performed on other extremophiles is necessary to expand our understanding of how microorganisms regulate pressure changes and how that governs their distribution on Earth.

## Data Availability Statement

The raw data supporting the conclusion of this article will be made available by the authors, without undue reservation, to any qualified researcher.

## Author Contributions

GO, AC, and KR designed this research project, analyzed the data. GO performed the experiments and collected the data, wrote the original draft. KR and AC edited and wrote sections of the manuscript. All authors contributed to revisions of the manuscript, tables, and figures and approved the submitted version.

## Conflict of Interest

The authors declare that the research was conducted in the absence of any commercial or financial relationships that could be construed as a potential conflict of interest.

## References

[B1] Abe F. Horikoshi K. (2001). The biotechnological potential of piezophiles. *Trends Biotechnol.* 19 102–108. 10.1016/S0167-7799(00)01539-0 11179803

[B2] Abe F. Kato C. Horikoshi K. (1999). Pressure-regulated metabolism in microorganisms. *Trends Microbiol.* 7 447–453. 10.1016/S0966-842X(99)01608-X 10542424

[B3] Achenbach-Richter L. Stetter K. O. Woese C. R. (1987). A possible biochemical missing link among archaebacteria. *Nature* 327 348–349. 10.1038/327348a0 11540893

[B4] Alain K. Marteinsson V. Miroshnichenko M. L. Bonch-osmolovskaya E. A. Prieur D. Birrien J. (2002). *Marinitoga piezophilia* sp. nov., a rod-shaped, thermo-piezophilic bacterium isolated under high hydrostatic pressure from a deep-sea hydrothermal vent. *Int. J. Syst. Evol. Microbiol.* 52 1331–1339. 10.1099/ijs.0.02068-0 12148648

[B5] Alazard D. Dukan S. Urios A. Verhé F. Bouabida N. Morel F. (2003). *Desulfovibrio hydrothermalis* sp. nov., a novel sulfate-reducing bacterium isolated from hydrothermal vents. *Int. J. Syst. Evol. Microbiol.* 53 173–178. 10.1099/ijs.0.02323-0 12656169

[B6] Balch W. E. Fox G. E. Magrum L. J. Woese C. R. Wolfe R. S. (1979). Methanogens: reevaluation of a unique biological group. *Microbiolol. Rev.* 43 260–296. 10.1016/j.watres.2010.10.010 390357 PMC281474

[B7] Bale S. J. Goodman K. Rochelle P. A. Marchesi J. R. Fry J. C. Weightman A. J. (1997). *Desulfovibrio profundus* sp. nov., a novel barophilic sulfate-reducing bacterium from deep sediment layers in the Japan sea. *Int. J. Syst. Bacteriol.* 47 515–521. 10.1099/00207713-47-2-515 9103642

[B8] Bar-On Y. M. Phillips R. Milo R. (2018). The biomass distribution on Earth. *Proc. Natl. Acad. Sci. U.S.A.* 115 6506–6511. 10.1073/pnas.1711842115 29784790 PMC6016768

[B9] Bartlett D. H. (2002). Pressure effects on in vivo microbial processes. *Biochim. Biophys. Acta* 1595 367–381. 10.1016/S0167-4838(01)00357-0 11983409

[B10] Beeder J. Nilsen R. K. Rosnes J. T. Torsvik T. Lien T. (1994). Archaeoglobus fulgidus isolated from hot North Sea oil field waters. *Appl. Environ. Microbiol.* 60 1227–1231. 10.1128/aem.60.4.1227-1231.1994 16349231 PMC201463

[B11] Bernhardt G. Jaenicke R. Lüdemann H. D. (1987). High-pressure equipment for growing methanogenic microorganisms on gaseous substrates at high temperature. *Appl. Environ. Microbiol.* 53 1876–1879. 10.1126/science.1086823 16347413 PMC204017

[B12] Bernhardt G. Jaenicke R. Lüdemann H. D. König H. Stetter K. O. (1988). High pressure enhances the growth rate of the thermophilic archaebacterium Methanococcus thermolithotrophicus without extending its temperature range. *Appl. Environ. Microbiol.* 54 1258–1261. 10.1128/aem.54.5.1258-1261.1988 16347635 PMC202636

[B13] Bianchi A. Garcin J. Tholosan O. (1999). A high-pressure serial sampler to measure microbial activity in the deep sea. *Deep-Sea Res. Part I* 46 2129–2142. 10.1016/S0967-0637(99)00039-4

[B14] Birrien J. L. Zeng X. Jebbar M. Cambon-Bonavita M. A. Quérellou J. Oger P. (2011). *Pyrococcus yayanosii* sp. nov., an obligate piezophilic hyperthermophilic archaeon isolated from a deep-sea hydrothermal vent. *Int. J. Syst. Evol. Microbiol.* 61 2827–2831. 10.1099/ijs.0.024653-0 21239564

[B15] Blöchl E. Rachel R. Burggraf S. Hafenbradl D. Jannasch H. W. Stetter K. O. (1997). *Pyrolobus fumarii*, gen. and sp. nov., represents a novel group of archaea, extending the upper temperature limit for life to 113 degrees C. *Extremophiles* 1 14–21. 10.1007/s007920050010 9680332

[B16] Boonyaratanakornkit B. Córdova J. Park C. B. Clark D. S. (2006). Pressure affects transcription profiles of *Methanocaldococcus jannaschii* despite the absence of barophilic growth under gas-transfer limitation. *Environ. Microbiol.* 8 2031–2035. 10.1111/j.1462-2920.2006.01083.x 17014501

[B17] Brysch K. Schneider C. Fuchs G. Widdel F. (1987). Lithoautotrophic growth of sulfate-reducing bacteria, and description of *Desulfobacterium autotrophicum* gen. nov., sp. nov. *Arch. Microbiol.* 148 264–274. 10.1007/BF00456703

[B18] Burggraf S. Jannasch H. W. Nicolaus B. Stetter K. O. (1990). *Archaeoglobus profundus* sp. nov., represents a new species within the sulfate-reducing archaebacteria. *Syst. Appl. Microbiol.* 13 24–28. 10.1016/S0723-2020(11)80176-1

[B19] Cario A. Jebbar M. Thiel A. Kervarec N. Oger P. M. (2016). Molecular chaperone accumulation as a function of stress evidences adaptation to high hydrostatic pressure in the piezophilic archaeon Thermococcus barophilus. *Sci. Rep.* 6 1–8. 10.1038/srep29483 27378270 PMC4932500

[B20] Cario A. Oliver G. C. Rogers K. L. (2019). Exploring the deep marine biosphere: challenges, innovations, and opportunities. *Front. Earth Sci.* 7:225 10.3389/feart.2019.00225

[B21] Charlesworth J. Burns B. P. (2016). Extremophilic adaptations and biotechnological applications in diverse environments. *AIMS Microbiol.* 2 251–261. 10.3934/microbiol.2016.3.251

[B22] Dalmasso C. Oger P. Selva G. Courtine D. L’Haridon S. Garlaschelli A. (2016). *Thermococcus piezophilus* sp. nov., a novel hyperthermophilic and piezophilic archaeon with a broad pressure range for growth, isolated from a deepest hydrothermal vent at the Mid-Cayman Rise. *Syst. Appl. Microbiol.* 39 440–444. 10.1016/j.syapm.2016.08.003 27638197

[B23] Donaldson E. C. Knapp R. M. Yen T. F. Chilingarian G. V. (1989). The subsurface environment. *Dev. Petrol. Sci.* 22 15–36. 10.1016/S0376-7361(09)70090-1

[B24] Erauso G. Reysenbach A. L. Godfroy A. Meunier J. R. Crump B. Partensky F. (1993). *Pyrococcus abyssi* sp. nov., a new hyperthermophilic archaeon isolated from a deep-sea hydrothermal vent. *Arch. Microbiol.* 160 338–349. 10.1007/BF00252219

[B25] Fang J. Zhang L. Bazylinski D. A. (2010). Deep-sea piezosphere and piezophiles: geomicrobiology and biogeochemistry. *Trends Microbiol.* 18 413–422. 10.1016/j.tim.2010.06.006 20663673

[B26] Fardeau M.-L. Goulhen F. Bruschi M. Khelifi N. Cayol J.-L. Ignatiadis I. (2009). Archaeoglobus fulgidus and Thermotoga efii, thermophilic isolates from deep geothermal water of the Paris Basin. *Geomicrobiol. J.* 26 119–130. 10.1080/01490450802674970

[B27] Fichtel K. Logemann J. Fichtel J. Rullkötter J. Cypionka H. Engelen B. (2015). Temperature and pressure adaptation of a sulfate reducer from the deep subsurface. *Front. Microbiol.* 6:1078. 10.3389/fmicb.2015.01078 26500624 PMC4594026

[B28] Garel M. Bonin P. Martini S. Guasco S. Roumagnac M. Bhairy N. (2019). Pressure-retaining sampler and high-pressure systems to study deep-sea microbes under in situ conditions. *Front. Microbiol.* 10:453. 10.3389/fmicb.2019.00453 31024462 PMC6465632

[B29] Gieg L. M. Davidova I. A. Duncan K. E. Suflita J. M. (2010). Methanogenesis, sulfate reduction and crude oil biodegradation in hot Alaskan oilfields. *Environ. Microbiol.* 12 3074–3086. 10.1111/j.1462-2920.2010.02282.x 20602630

[B30] Gieg L. M. Jack T. R. Foght J. M. (2011). Biological souring and mitigation in oil reservoirs. *Appl. Microbiol. Biotechnol.* 92 263–282. 10.1007/s00253-011-3542-6 21858492

[B31] Gittel A. Sørensen K. B. Skovhus T. L. Ingvorsen K. Schramm A. (2009). Prokaryotic community structure and sulfate reducer activity in water from high-temperature oil reservoirs with and without nitrate treatment. *Appl. Environ. Microbiol.* 75 7086–7096. 10.1128/AEM.01123-09 19801479 PMC2786513

[B32] Goldhaber M. B. (2003). Sulfur-rich sediments. *Treatise Geochem.* 7–9 257–288. 10.1016/B0-08-043751-6/07139-5

[B33] Hartzell P. Reed D. W. (2006). The genus *Archaeoglobus*. *Prokaryotes* 3 82–100. 10.1007/0-387-30747-8

[B34] Hartzell P. L. Millstein J. Lapaglia C. (1999). Biofilm formation in hyperthermophilic archaea. *Methods Enzymol.* 310 335–349. 10.1016/S0076-6879(99)10027-2 10547803

[B35] Hei D. J. Clark D. S. (1994). Pressure stabilization of proteins from extreme thermophiles. *Appl. Environ. Microbiol.* 60 932–939. 10.1038/nature06899 16349220 PMC201413

[B36] Hinrichs K.-U. Boetius A. (2002). “The anaerobic oxidation of methane: new insights in microbial ecology and biogeochemistry,” in *Ocean Margin Systems*, eds Wefer G. Billett D. Hebbeln D. Jørgensen B. B. Schlüter M. van Weering T. C. E. (Berlin: Springer), 457–477. 10.1007/978-3-662-05127-6_28

[B37] Hocking W. P. Stokke R. Roalkvam I. Steen I. H. (2014). Identification of key components in the energy metabolism of the hyperthermophilic sulfate-reducing archaeon *Archaeoglobus fulgidus* by transcriptome analyses. *Front. Microbiol.* 5:95. 10.3389/fmicb.2014.00095 24672515 PMC3949148

[B38] Holden J. F. Baross J. A. (1993). Enhanced thermotolerance and temperature-induced changes in protein composition in the hyperthermophilic archaeon ES4. *J. Bacteriol.* 175 2839–2843. 10.1128/jb.175.10.2839-2843.1993 8491704 PMC204599

[B39] Holden J. F. Baross J. A. (1995). Enhanced thermotolerance by hydrostatic pressure in the deep-sea hyperthermophile Pyrococcus strain ES4. *FEMS Microbiol. Ecol.* 18 27–33. 10.1016/0168-6496(95)00037-B

[B40] Huber H. Huber R. Stetter K. O. (2006). Thermoproteales. *Prokaryotes* 3 10–22. 10.1007/0-387-30743-5_2

[B41] Huber H. Jannasch H. Rachel R. Fuchs T. Stetter K. O. (1997). *Archaeoglobus veneficus* sp nov, a novel facultative chemolithoautotrophic hyperthermophilic sulfite reducer, isolated from abyssal black smokers. *Syst. Appl. Microbiol.* 20 374–380. 10.1016/S0723-2020(97)80005-7

[B42] Huber R. Woese C. R. Langworthy T. A. Fricke H. Stetter K. O. (1989). *Thermosipho africanus* gen. nov., represents a new genus of thermophilic eubacteria within the “Thermotogales.”. *Syst. Appl. Microbiol.* 12 32–37. 10.1016/S0723-2020(89)80037-2

[B43] Jaenicke R. (1983). Biochemical processes under high hydrostatic pressure - Physico-chemical approaches to barosensitivity. *Naturwissenschaften* 70 332–341. 10.1007/BF00444207

[B44] Jannasch H. W. Taylor C. D. (1984). Deep-sea microbiology. *Annu. Rev. Microbiol.* 38 487–514. 6437324 10.1146/annurev.mi.38.100184.002415

[B45] Jannasch H. W. Wirsen C. O. (1973). Deep-sea microorganisms: in situ response to nutrient enrichment. *Science* 180 641–643. 10.1126/science.180.4086.641 17774289

[B46] Jannasch H. W. Wirsen C. O. Molyneaux S. J. Langworthy T. A. (1992). Comparative physiological studies on hyperthermophilic archaea isolated from Deep-sea hot vents with emphasis on Pyrococcus strain GB-D. *Appl. Environ. Microbiol.* 58 3472–3481. 16348799 10.1128/aem.58.11.3472-3481.1992 PMC183131

[B47] Jebbar M. Franzetti B. Girard E. Oger P. (2015). Microbial diversity and adaptation to high hydrostatic pressure in deep-sea hydrothermal vents prokaryotes. *Extremophiles* 19 721–740. 10.1007/s00792-015-0760-3 26101015

[B48] Joye S. B. Boetius A. Orcutt B. N. Montoya J. P. Schulz H. N. Erickson M. J. (2004). The anaerobic oxidation of methane and sulfate reduction in sediments from Gulf of Mexico cold seeps. *Chem. Geol.* 205 219–238. 10.1016/j.chemgeo.2003.12.019

[B49] Kallmeyer J. Boetius A. (2004). Effects of temperature and pressure on sulfate reduction and anaerobic oxidation of methane in hydrothermal sediments of Guaymas Basin. *Appl. Environ. Microbiol.* 70 1231–1233. 10.1128/AEM.70.2.1231-1233.2004 14766611 PMC348843

[B50] Kallmeyer J. Ferdelman T. G. Jansen K. H. Jørgensen B. B. (2003). A high-pressure thermal gradient block for investigating microbial activity in multiple deep-sea samples. *J. Microbiol. Methods* 55 165–172. 10.1016/S0167-7012(03)00138-6 14500008

[B51] Kaneshiro S. M. Clark D. S. (1995). Pressure effects on the composition and thermal behavior of lipids from the deep-sea thermophile Methanococcus jannaschii. *J. Bacteriol.* 177 3668–3672. 10.1128/jb.177.13.3668-3672.1995 7601829 PMC177081

[B52] Kato C. Nogi Y. Arakawa S. (2008). “Isolation, cultivation, and diversity of deep-sea piezophiles,” in *High-Pressure Microbiology*, eds Michiels C. Bartlett D. H. Aertsen A. (Washington, DC: ASM Press), 203–218.

[B53] Kepner R. L. Pratt J. R. (1994). Use of fluorochromes for direct enumeration of total bacteria in environmental samples: past and present. *Microbiol. Rev.* 58 603–615. 10.1128/mmbr.58.4.603-615.1994 7854248 PMC372983

[B54] Khelaifia S. Fardeau M. L. Pradel N. Aussignargues C. Garel M. Tamburini C. (2011). *Desulfovibrio piezophilus* sp. nov., a piezophilic, sulfate-reducing bacterium isolated from wood falls in the Mediterranean Sea. *Int. J. Syst. Evol. Microbiol.* 61 2706–2711. 10.1099/ijs.0.028670-0 21169465

[B55] Khelifi N. Amin Ali O. Roche P. Grossi V. Brochier-Armanet C. Valette O. (2014). Anaerobic oxidation of long-chain n-alkanes by the hyperthermophilic sulfate-reducing archaeon. *Archaeoglobus fulgidus*. *ISME J.* 8 3057–3060. 10.1038/ismej.2014.58 24763368 PMC4992073

[B56] Khelifi N. Grossi V. Hamdi M. Dolla A. Tholozan J. L. Ollivier B. (2010). Anaerobic oxidation of fatty acids and alkenes by the hyperthermophilic sulfate-reducing archaeon *Archaeoglobus fulgidus*. *Appl. Environ. Microbiol.* 76 3057–3060. 10.1128/AEM.02810-09 20305028 PMC2863424

[B57] Klenk H. P. Clayton R. A. Tomb J. F. White O. Nelson K. E. Ketchum K. A. (1997). The complete genome sequence of the hyperthermophilic, sulphate-reducing archaeon *Archaeoglobus fulgidus*. *Nature* 390 364–370. 10.1038/37052 9389475

[B58] Kusube M. Kyaw T. S. Tanikawa K. Chastain R. A. Hardy K. M. Cameron J. (2017). *Colwellia marinimaniae* sp. nov., a hyperpiezophilic species isolated from an amphipod within the challenger deep. Mariana Trench. *Int. J. Syst. Evol. Microbiol.* 67 824–831. 10.1099/ijsem.0.001671 27902293

[B59] Lauro F. M. Bartlett D. H. (2007). Prokaryotic lifestyles in deep sea habitats. *Extremophiles* 12 15–25. 10.1007/s00792-006-0059-5 17225926

[B60] Lauro F. M. Chastain R. A. Blankenship L. E. Yayanos A. A. Bartlett D. H. (2007). The unique 16S rRNA genes of piezophiles reflect both phylogeny and adaptation. *Appl. Environ. Microbiol.* 73 838–845. 10.1128/aem.01726-06 17158629 PMC1800765

[B61] L’Haridon S. Reysenbach A.-L. Glenat R. Prieur D. Jeanthon C. (1995). Hot subterranean bioshphere in a continental oil reservoir. *Nature* 377 223–224. 10.1038/377223a0 8588738

[B62] Liu R. Wang L. Wei Y. Fang J. (2018). The hadal biosphere: Recent insights and new directions. *Deep-Sea Res. Part II* 155 11–18. 10.1016/j.dsr2.2017.04.015

[B63] Marteinsson V. T. Birrien J. L. Raguenes G. da Costa M. S. Prieur D. (1999). Isolation and characterization of Thermus thermophilus Gy1211 from a deep-sea hydrothermal vent. *Extremophiles* 3 247–251. 10.1007/s007920050123 10591014

[B64] Marteinsson V. T. Moulin P. Birrien J. Gambacorta A. Vernet M. Prieur D. (1997). Physiological responses to stress conditions and barophilic behavior of the hyperthermophilic vent archaeon pyrococcus physiological responses to stress conditions and barophilic behavior of the hyperthermophilic vent archaeon *Pyrococcus abyssi*. *Appl. Environ. Microbiol.* 63 1230–1236. 10.1128/aem.63.4.1230-1236.1997 16535565 PMC1389543

[B65] McNichol J. Sylva S. P. Thomas F. Taylor C. D. Sievert S. M. Seewald J. S. (2016). Assessing microbial processes in deep-sea hydrothermal systems by incubation at in situ temperature and pressure. *Deep-Sea Res. Part I* 115 221–232. 10.1016/j.dsr.2016.06.011

[B66] Meulepas R. J. W. Jagersma C. G. Khadem A. F. Stams A. J. M. Lens P. N. L. (2010). Effect of methanogenic substrates on anaerobic oxidation of methane and sulfate reduction by an anaerobic methanotrophic enrichment. *Appl. Microbiol. Biotechnol.* 87 1499–1506. 10.1007/s00253-010-2597-0 20445975 PMC2892604

[B67] Michels P. C. Hei D. Clark D. S. (1996). Enzymes and proteins from hyperthermophilic microorganisms. *Adv. Protein Chem.* 48 341–376. 10.1016/S0065-3233(08)60366-6 8791629

[B68] Michoud G. Jebbar M. (2016). High hydrostatic pressure adaptive strategies in an obligate piezophile *Pyrococcus yayanosii*. *Scientific Reports* 6 1–10. 10.1038/srep27289 27250364 PMC4890121

[B69] Miller J. A. Y. F. Shah N. N. Nelson C. M. Ludlow J. A. N. M. Clark D. S. (1988). Pressure and temperature effects on growth and methane production of the extreme thermophile *Methanococcus jannaschii*. *Appl. Environ. Microbiol.* 54 3039–3042. 10.1128/aem.54.12.3039-3042.1988 16347794 PMC204424

[B70] Mori K. Maruyama A. Urabe T. Suzuki K. I. Hanada S. (2008). *Archaeoglobus infectus* sp. nov., a novel thermophilic, chemolithoheterotrophic archaeon isolated from a deep-sea rock collected at Suiyo Seamount. Izu-Bonin Arc, western Pacific Ocean. *Int. J. Syst. Evol. Microbiol.* 58 810–816. 10.1099/ijs.0.65422-0 18398174

[B71] Nakagawa S. Takai K. Inagaki F. Chiba H. Ishibashi J. I. Kataoka S. (2005). Variability in microbial community and venting chemistry in a sediment-hosted backarc hydrothermal system: impacts of subseafloor phase-separation. *FEMS Microbiol. Ecol.* 54 141–155. 10.1016/j.femsec.2005.03.007 16329980

[B72] Nercessian O. Reysenbach A. L. Prieur D. Jeanthon C. (2003). Archaeal diversity associated with in situ samplers deployed on hydrothermal vents on the East Pacific Rise. *Environ. Microbiol.* 5 492–502. 10.1046/j.1462-2920.2003.00437.x 12755716

[B73] Nogi Y. Kato C. Horikoshi K. (1998). *Moritella japonica* sp. nov., a novel barophilic bacterium isolated from a Japan Trench sediment. *J. Gen. Appl. Microbiol.* 44 289–295. 10.2323/jgam.44.289 12501424

[B74] Oger P. M. Jebbar M. (2010). The many ways of coping with pressure. *Res. Microbiol.* 161 799–809. 10.1016/j.resmic.2010.09.017 21035541

[B75] Park C. B. Clark D. S. (2002). Rupture of the cell envelope by decompression of the deep-sea methanogen *Methanococcus jannaschii*. *Appl. Environ. Microbiol.* 68 1458–1463. 10.1128/AEM.68.3.1458 11872502 PMC123755

[B76] Peoples L. M. Bartlett D. H. (2017). “Ecogenomics of deep-ocean microbial bathytypes,” in *Microbial Ecology of Extreme Environments*, eds Chénard C. Lauro F. M. (Berlin: Springer), 7–50. 10.1007/978-3-319-51686-8

[B77] Picard A. Daniel I. (2013). Pressure as an environmental parameter for microbial life - A review. *Biophys. Chem.* 183 30–41. 10.1016/j.bpc.2013.06.019 23891571

[B78] Pinder D. (2001). Offshore oil and gas: Global resource knowledge and technological change. *Ocean Coast. Manag.* 44 579–600. 10.1016/S0964-5691(01)00070-9

[B79] Prieur D. Marteinsson V. T. (1998). Prokaryotes living under elevated hydrostatic pressure. *Adv. Biochem. Eng. Biotechnol.* 61 23–35. 10.1007/bfb0102288

[B80] Rabus R. Hansen T. A. Widdel F. (2013). “Dissimilatory sulfate- and sulfur-reducing prokaryotes,” in *The Prokaryotes: Prokaryotic Physiology and Biochemistry*, eds Rosenberg E. (Berlin: Springer), 309–404. 10.1007/978-3-642-30141-4_70

[B81] Reveillaud J. Reddington E. McDermott J. Algar C. Meyer J. L. Sylva S. (2016). Subseafloor microbial communities in hydrogen-rich vent fluids from hydrothermal systems along the Mid-Cayman Rise. *Environ. Microbiol.* 18 1970–1987. 10.1111/1462-2920.13173 26663423 PMC5021209

[B82] Reysenbach A. L. Holm N. G. Hershberger K. Prieur D. Jeanthon C. (1998). In search of a subsurface biosphere at a slow-spreading ridge. *Proc. Ocean Dril. Prog.* 158 355–360. 10.2973/odp.proc.sr.158.229.1998

[B83] Reysenbach A. L. Longnecker K. Kirshtein J. (2000). Novel bacterial and archaeal lineages from an in situ growth chamber deployed at a mid-atlantic ridge hydrothermal vent. *Appl. Environ. Microbiol.* 66 3798–3806. 10.1128/AEM.66.9.3798-3806.2000 10966393 PMC92223

[B84] Schrenk M. O. Kelley D. S. Delaney J. R. Baross J. A. (2003). Incidence and diversity of microorganisms within the walls of an active deep-sea sulfide chimney. *Appl. Environ. Microbiol.* 69 3580–3592. 10.1128/AEM.69.6.3580-3592.2003 12788766 PMC161516

[B85] Scoma A. Garrido-Amador P. Nielsen S. D. Røy H. Kjeldsen K. U. (2019). The polyextremophilic bacterium Clostridium paradoxum attains piezophilic traits by modulating its energy metabolism and cell membrane composition. *Appl. Environ. Microbiol.* 85 1–14. 10.1128/AEM.00802-19 PMC6643245 31126939

[B86] Steinsbu B. O. Thorseth I. H. Nakagawa S. Inagaki F. Lever M. A. Engelen B. (2010). *Archaeoglobus sulfaticallidus* sp. nov., a thermophilic and facultatively lithoautotrophic sulfate-reducer isolated from black rust exposed to hot ridge flank crustal fluids. *Int. J. Syst. Evol. Microbiol.* 60 2745–2752. 10.1099/ijs.0.016105-0 20061497

[B87] Stetter K. (1992). The genus *Archaeoglobus*. *Prokaryotes* 2 707–711.

[B88] Stetter K. O. (1988). *Archaeoglobus fulgidus* gen. nov., sp. nov.: a new taxon of extremely thermophilic archaebacteria. *Syst. Appl. Microbiol.* 10 172–173. 10.1016/S0723-2020(88)80032-8

[B89] Stetter K. O. Fiala G. Huber G. Huber R. Segerer A. (1990). Hyperthermophilic microorganisms. *FEMS Microbiol. Lett.* 75 117–124. 10.1016/0378-1097(90)90526-V 24414408

[B90] Stetter K. O. Huber R. Blöchl E. Kurr M. Eden R. D. Fielder M. (1993). Hyperthermophilic archaea are thriving in deep North Sea and Alaskan oil reservoirs. *Nature* 365 743–745. 10.1038/365743a0

[B91] Stetter K. O. Lauerer G. Thomm M. Neuner A. (1987). Isolation of extremely thermophilic sulfate reducers: evidence for a novel branch of archaebacteria. *Science* 236 822–824. 10.1126/science.236.4803.822 17777850

[B92] Straube W. L. O’Brien M. Davis K. Colwell R. R. (1990). Ezymatic profiles of 11 barophilic bacteria under in situ conditions: Evidence for pressure modulation of phenotype. *Appl. Environ. Microbiol.* 56 812–814. 10.1128/aem.56.3.812-814.1990 2317048 PMC183426

[B93] Sun M. M. Clark D. S. (2001). “Pressure effects on activity and stability of hyperthermophilic enzymes,” in *Methods in Enzymology*, eds Abelson J. Simon M. Verdine G. Pyle A. (Cambridge, MA: Academic Press). 10.1016/s0076-6879(01)34479-8 11398475

[B94] Takai K. Horikoshi K. (1999). Genetic diversity of archaea in deep-sea hydrothermal vent environments. *Genetics* 152 1285–1297. 10.1016/s0723-2020(87)80053-x 10430559 PMC1460697

[B95] Takai K. Miyazaki M. Hirayama H. Nakagawa S. Querellou J. Godfroy A. (2009). Isolation and physiological characterization of two novel, piezophilic, thermophilic chemolithoautotrophs from a deep-sea hydrothermal vent chimney. *Environ. Microbiol.* 11 1983–1997. 10.1111/j.1462-2920.2009.01921.x 19689705

[B96] Takai K. Nakamura K. Toki T. Tsunogai U. Miyazaki M. Miyazaki J. (2008). Cell proliferation at 122°C and isotopically heavy CH4 production by a hyperthermophilic methanogen under high-pressure cultivation. *Proc. Natl. Acad. Sci. U.S.A.* 105 10949–10954. 10.1073/pnas.0712334105 18664583 PMC2490668

[B97] Tasumi E. Yanagawa K. Miyazaki J. Takai K. (2015). “In vitro high-pressure incubation and activity measurement of deep-sea methanogenic archaea,” in *Hydrocarbon and Lipid Microbiology Protocols*, eds McGenity T. J. (Berlin: Springer), 51–64. 10.1007/8623_2015_111

[B98] Teske A. Hinrichs K. Edgcomb V. Gomez A. D. V. Kysela D. Sylva S. P. (2002). Microbial diversity of hydrothermal sediments in the guaymas basin: evidence for anaerobic methanotrophic communities. *Am. Soc. Microbiol.* 68 1994–2007. 10.1128/AEM.68.4.1994 PMC123873 11916723

[B99] Thauer R. K. Kunow J. (1995). “Sulfate-Reducing Archaea,” in *Sulfate-Reducing Bacteria*, ed. Barton L. L. (New York: Plenum Press), 33–45.

[B100] Vannier P. Michoud G. Oger P. Marteinsson V. P. Jebbar M. (2015). Genome expression of *Thermococcus barophilus* and *Thermococcus kodakarensis* in response to different hydrostatic pressure conditions. *Res. Microbiol.* 166 717–725. 10.1016/j.resmic.2015.07.006 26239966

[B101] Vezzi A. (2005). Life at Depth: *Photobacterium profundum* genome sequence and expression analysis. *Science* 307 1459–1461. 10.1126/science.1103341 15746425

[B102] Welch T. J. Farewell A. Neidhardt F. C. Bartlett D. H. (1993). Stress response of *Escherichia coli* to elevated hydrostatic pressure. *J. Bacteriol.* 175 7170–7177. 10.1128/jb.175.22.7170-7177.1993 8226663 PMC206858

[B103] Whitman W. B. Coleman D. C. Wiebe W. J. (1998). Prokaryotes: The unseen majority. *Proc. Natl. Acad. Sci. U.S.A.* 95 6578–6583. 10.1073/pnas.95.12.6578 9618454 PMC33863

[B104] Williamson A. J. Carlson H. K. Kuehl J. V. Huang L. L. Iavarone A. T. Deutschbauer A. (2018). Dissimilatory sulfate reduction under high pressure by *Desulfovibrio alaskensis* G20. *Front. Microbiol.* 9:1465. 10.3389/fmicb.2018.01465 30050504 PMC6052904

[B105] Yayanos A. A. (1995). Microbiology to 10,500 meters in the deep sea. *Annu. Rev. Microbiol.* 49 777–805. 10.1146/annurev.micro.49.1.777 8561479

[B106] Yayanos A. A. (2001). 30 Deep-sea piezophilic bacteria. *Methods Microbiol.* 30 615–637. 10.1016/s0580-9517(01)30065-x

[B107] Yayanos A. A. Dietz A. S. Boxtel V. (1982). Dependance of reproduction rate on pressure as a hallmark of deep-sea bacteria. *Appl. Environ. Microbiol.* 44 1356–1361. 10.1128/aem.44.6.1356-1361.1982 16346153 PMC242196

[B108] Yu L. Zhou Z. Wei S. Xu X. Wang Q. Xu G. (2019). *Marinomonas piezotolerans* sp. nov., isolated from deep-sea sediment of the Yap Trench, Pacific Ocean. *Int. J. Syst. Evol. Microbiol.* 69 739–744. 10.1099/ijsem.0.003227 30648946

[B109] Zellner G. Stackebrandt E. Kneifel H. Messner P. Sleytr U. B. de Macario E. C. (1989). Isolation and characterization of a thermophilic, sulfate reducing archaebacterium. *Archaeoglobus fulgidus* Strain Z. *Syst. Appl. Microbiol.* 11 151–160. 10.1016/S0723-2020(89)80055-4

[B110] Zeng X. Birrien J.-L. Fouquet Y. Cherkashov G. Jebbar M. Querellou J. (2009). Pyrococcus CH1, an obligate piezophilic hyperthermophile: extending the upper pressure-temperature limits for life. *ISME J.* 3 873–876. 10.1038/ismej.2009.21 19295639

[B111] Zhang Y. Li X. Xiao X. Bartlett D. H. (2015). Current developments in marine microbiology: High-pressure biotechnology and the genetic engineering of piezophiles. *Curr. Opin. Biotechnol* 33 157–164. 10.1016/j.copbio.2015.02.013 25776196

[B112] Zhao W. Zeng X. Xiao X. (2015). *Thermococcus eurythermalis* sp. nov., a conditional piezophilic, hyperthermophilic archaeon with a wide temperature range for growth, isolated from an oil-immersed chimney in the Guaymas Basin. *Int. J. Syst. Evol. Microbiol.* 65 30–35. 10.1099/ijs.0.067942-0 25288278

[B113] Zobell C. E. Oppenheimer C. H. (1950). Some effects of hydrostatic pressure on the multiplication and morphology of marine bacteria. *J. Bacteriol.* 60 771–781. 10.1128/jb.60.6.771-781.1950 14824070 PMC385948

