# Supplementary material for: Rate and Extent of Growth of a Model Extremophile, Archaeoglobus fulgidus, Under High Hydrostatic Pressures
Source: Front Microbiol. 2020 Jun 12;11:1023. doi: 10.3389/fmicb.2020.01023 (PMC7303961; doi:10.3389/fmicb.2020.01023)
Supplement: Supplementary file 1 [file Data_Sheet_1.PDF]

Supplementary Material

1 *A. fulgidus* decompression and repressurization tests

*S*ince traditional high-pressure batch cultivation of microbial species usually requires short periods of decompression - and cooling for thermophiles - when subsampling (Park and Clark, 2002), a suite of experiments was conducted to test if these methods affected the observed growth yields. To assess if sample decompression affected *A. fulgidus* growth, cell densities from cells pressurized and decompressed once were compared to cell densities from cells decompressed and repressurized multiple times. The experimental design made use of four static pressure vessels, each containing syringes with growth medium inoculated from the same triplicate precultures, and pressurized at the same time. One vessel was decompressed without sampling after six hours, and repressurized. This vessel was then decompressed with sampling at 12 hours, 24 hours and 36 hours after inoculation. The second vessel was decompressed once after 12 hours of growth and cell densities were compared to cells grown in the first vessel after 12 hours of growth. Next, the third vessel was decompressed after 24 hours and the fourth vessel was decompressed after 36 hours respectively and again cell densities were compared to those in the first vessel that experiences multiple cycles of decompression and repressurization (Supplementary Figure 1). These decompression tests were conducted from 10 – 60 MPa at 10 MPa increments.

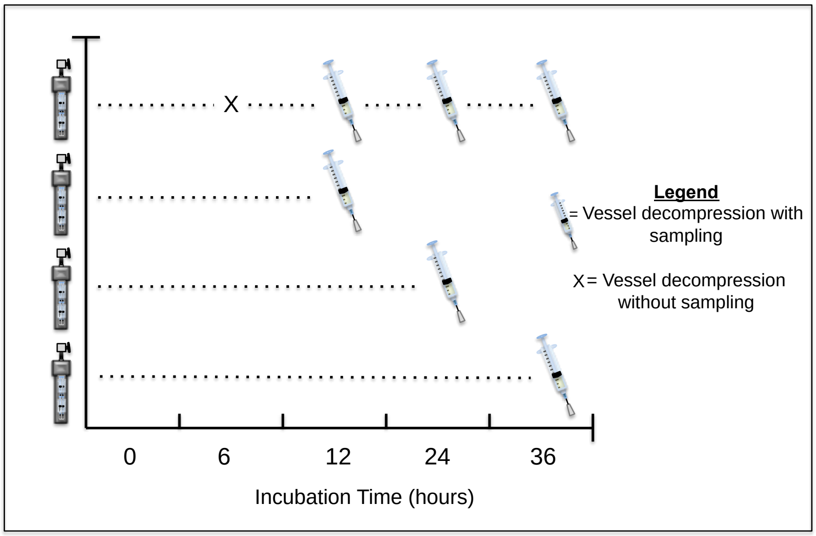

Supplementary Figure S1. Decompression, repressurization, and sampling schedule for the suite of experiments performed to test if sample decompression affected cell densities.

2 Direct counts from DAPI staining versus Thoma-Chamber

To compare the two methods used for direct cell counts, *A. fulgidus was grown in a heterotrophic medium in* triplicate at 0.3 MPa and 83°C in Balch tubes, and each triplicate experiment was counted by both DAPI staining and Thoma Chamber methods. Here, the sulfate and lactate rich heterotrophic growth medium consisted of (per liter): 0.34g KCl, 3.45g $MgSO_4•7H_2O$, 4.00g $MgCl•6H_2O$, 0.25g NH_2Cl, 0.14g $CaCl_2•2H_2O$, 0.14g $K_2HPO_4•3H_2O$, 18g NaCl, 0.002g $Fe(NH_4)_2(SO_4)•6H_2O$, 1.5g

sodium L-lactate ($NaC_3H_5O_3$), 1g yeast extract, 3.36g PIPES (piperazine-*N*,*N'*bis[2-ethanesulfonic acid]), 0.1 mL Resazurin (0.1% solution), and 10 mL trace element solution (DSMZ, medium 141). The pH was adjusted to 6.7. To obtain anoxia, the medium was boiled under an N_2 atmosphere and 10 mL of medium was transferred into individual N_2-flushed Balch tubes, the tubes were sealed with butyl rubber stoppers and crimp-sealed prior to autoclaving. The medium was further reduced with 2.5 % (w/v) $Na_2S \cdot 9H_2O$ stock solution to a final concentration of 1 mM, and then inoculated with 3% (v/v) logarithmic phase *A. fulgidus* cells from three separate precultures to a final cell concentration of ~7.7 x 10^6 cells/mL. Growth experiments carried out were similar to those described in section 2.2.

Subsamples were collected ever two to four hours for the first 24 hours after inoculation, then subsamples were taken 36 and 48 hours after inoculation. Each subsample was counted using both DAPI staining and Thoma Chamber techniques described in section 2.4. Figure S2 plots cell densities as a function of time for both counting methods, and at each time point the measured cell densities are within error of each other. A 2-sample independent, two tailed t-test was performed on the two data sets, calculated in Minitab®19. There was no significant difference, $t(76) = 0.40$, $p = .689$ or $p < .05$, between the two counting methods

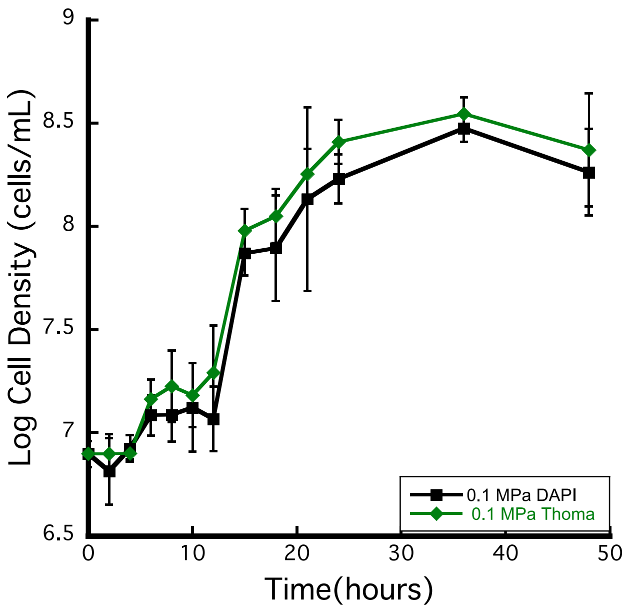

Supplementary Figure S2. Direct counts of *A. fulgidus* cells grown at 0.3 MPa in Balch tubes using DAPI staining (black squares) and Thoma Chamber (green diamonds) methods. Error bars represent the standard deviation from at least triplicate experiments.

3 *A. fulgidus* adaptation from heterotrophy to autotrophy

A. fulgidus was successfully adapted to grow from a heterotrophic metabolism to an autotrophic metabolism through a series of growth experiments and transfers. *A. fulgidus* VC-16 was received from the Deutsche Sammlung von Mikroorganismen und Zellkulturen GmbH (DSMZ, Braunschweig, Germany) and grown in a lactate- and sulfate-rich medium supplemented with yeast extract. The first adaption step was to decrease the amount of yeast extract from 2 g/L to 1 g/L in the growth medium and supplement *A. fulgidus* growth with 1% (v/v) vitamin solution (DSMZ medium 141). *A. fulgidus* was successfully grown and transferred three consecutive times to late exponential

phase, then was transferred into medium with 0.5 g/L yeast extract and 2% (v/v) vitamin solution. *A. fulgidus* was successfully grown in growth media with decreasing amounts of yeast extract and with at most 2% (v/v) vitamin solution until high cell density growth (~10^8 cells/mL) was observed without the addition of yeast extract. Next, *A. fulgidus* was adapted to grow without the vitamin solution in lactate- and sulfate-rich medium only.

A. fulgidus cells adapted to grow in the lactate- and sulfate-rich medium without a supplement were used to further adapt this strain to autotrophic growth with H_2 as an electron donor and thiosulfate as an electron acceptor, while fixing carbon from CO_2 into its biomass. Using consecutive transfers of cells, *A. fulgidus* was first inoculated into the autotrophic medium described in section 2.1 with the only amendment being the addition of 2% (v/v) vitamin solution (DSMZ medium 141). *A. fulgidus* was successfully grown autotrophically and after three consecutive transfers in the same medium with a vitamin supplement, it was transferred into medium without vitamins. Finally, *A. fulgidus* was successfully grown autotrophically without yeast extract or vitamins. Standard cellular stocks were made with these autotrophic-adapted *A. fulgidus* cells. *A. fulgidus* autotrophic HHP growth experiment pre-cultures were prepared from these standard cellular stocks.

4 Cellular aggregation

A. fulgidus cellular aggregation and clustering was observed in samples grown in headspace-free syringes at 0.1-50 MPa, but was not observed in samples grown in Balch tubes at near-ambient pressure conditions (0.3 MPa; Figure S3). Since aggregation was observed in both ambient pressure and high-pressure experiments performed in headspace-free syringes, pressure is not thought to be the initiating factor causing this physiological response. Further investigations are underway to determine the parameter(s) inducing this response.

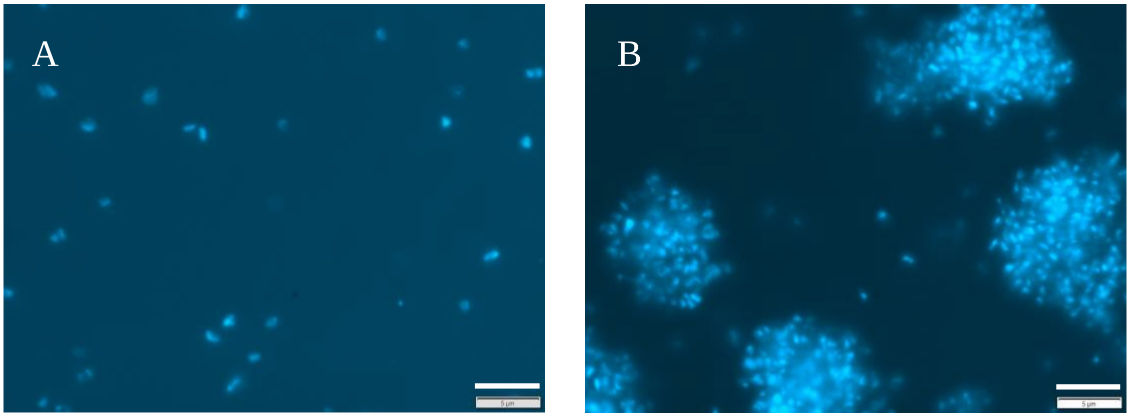

Supplementary Figure S3. DAPI stained heterotrophic *A. fulgidus* cells grown at 0.3 MPa in Balch tubes (A) and *A. fulgidus* cell clustering at 10 MPa in headspace-free syringes (B) after 36 hours. Samples were vortexed for 10 seconds before filtration with a dilution factor of 20x and bar is 5 µm for both A and B.

5 References

Park, C. B., & Clark, D. S. (2002). Rupture of the cell envelope by decompression of the deep-sea methanogen *Methanococcus jannaschii*. *Applied and Environmental Microbiology*, *68*(3), 1458–1463. doi: 10.1128/AEM.68.3.1458
